# Appropriate complementary feeding practice and associated factors among mothers having children aged 6–24 months in Debre Tabor Hospital, North West Ethiopia, 2016

**DOI:** 10.1186/s13104-019-4259-3

**Published:** 2019-04-08

**Authors:** Asrat Hailu Dagne, Kiber Temesgen Anteneh, Marta Berta Badi, Hadgay Hagos Adhanu, Mekonnen Assefa Ahunie, H/Mariam Demewozu Tebeje, Getie Lake Aynalem

**Affiliations:** School of Midwifery, University of Gondar, Gondar City, Ethiopia

**Keywords:** Appropriate, Infant and young children, Complementary feeding, Ethiopia

## Abstract

**Objective:**

This study was aimed to assess appropriate complementary feeding practice and associated factors among mothers having children aged 6–24 months in Debre Tabor Hospital, North West Ethiopia, 2016.

**Results:**

In this study, 37.2% of mothers had appropriate complementary feeding practice. Mothers’ level of education above grade 12 (AOR = 2.96, CI 1.2–7.62), husbands’ occupation (AOR = 4.01, CI 1.3–12.44), mothers ‘having exclusive breast feeding practice (AOR = 6.12, CI 3.04–12.3), health education about exclusive breast feeding during antenatal care visit (AOR = 5.59, CI 1.24–25.17) and advice on appropriate complementary feeding practice during antenatal care visit (AOR = 6.34, CI 1.5–26.91), and mothers who have got under 5 unit service due to infant and young children illness (AOR = 0.44, CI 0.22–0.89) were statistically significant variables for appropriate complementary feeding practice.

## Introduction

Complementary feeding practice is the gradual prolusion of soft food, semisolid and solid food starting from children aged 6 month. In addition, breast feeding and timely initiation of complementary feeding practice is very important [1]. Malnutrition reduces the immune system and causes frequent illness in children. It is one of the world’s problems particularly in developing countries [2–4].

Every year, there are more than ten million under five children deaths in the world from which 98% occurred in developing countries [5]. Ethiopia is one of the countries where infant and under 5 mortality rate is the highest (48 per 1000 live birth and 67 per 1000 live birth respectively) [6]. About 50% of child death is related to malnutrition which can be preventable through appropriate complementary feeding practice including breast feeding (BF). Breast milk (BM) and complementary feeding could prevent 13% and 6% of under 5 child mortality respectively [7]. Malnutrition continues a major health problem that in 2011, globally, 165 million under 5 children were stunted, 100 million were under weight and 52 million were wasted [8]. In Ethiopia, the rate of stunting, underweight and wasting among under 5 children were 40%, 25% and 9% respectively [9].

The magnitude of appropriate complementary feeding practice is unacceptably low in the studies conducted in different countries of the world like 32% in India [10], 24.1% in Bennin [11], 37% in Zambia [12], and 15.7% in Ghana [13]. These studies also indicated low minimum dietary diversity (MDD). Children are stunted, if infant and young children feeding practice (IYCFP) is inappropriate. Based on this, world health organization has developed a set of core indicators to assess infant and young children feeding (IYCF) practice and these indicators include both breast feeding and complementary feeding practice [14]. Minimum dietary diversity is one of the components of infant and young children feeding practice (IYCFP) and low dietary diversity is associated with stunting [2].

The magnitude of appropriate complementary feeding practice in Ethiopia is low which accounts 4.8% in 2011. There is evidence that shows appropriate complementary feeding practice reduces stunting and improves better health and growth [8, 15].

This study, therefore, has identified the magnitude of appropriate complementary feeding practice and associated factors among mothers having children aged 6–24 months in Debre Tabor Genenral Hospital.

## Main text

### Methods

#### Study design and setting

An institutional based cross sectional study design was conducted in Debre Tabor Hospital at expanded program of immunization and under five units from April 1 to July 30/2016. The study setting (Debre Tabor Hospital) is the only Hospital in South Gondar Zone. It serves a population of approximately 2.3 million people, including 55,596 residents living in the town of Debre Tabor, of whom 27,644 are men and 27,952 women. The hospital is found in Debre Tabor town, which is the capital of south Gondar zone, Amhara region.

#### Sample size and sampling techniques

Sample size was calculated using single population proportion formula using the following assumptions; desired precision (d) = 5%, Confidence level = 95% (Ζα/2 = 1.96 value) and 60.5% of the prevalence of complementary feeding practice in Hiwot Fana Hospital was considered. Hence, the calculated sample size by considering 10% non- response rate was 409.

Systematic random sampling technique was employed to select study participants. From the previous data of hospital, the expected number of study population during the study period was 1800. The Kth interval number approximates 4 and the first number was taken randomly between 1 and 4 that was 3, then the rest samples were taken every 4th digit intervals.

#### Data collection instrument and procedures

A semi-structured questionnaire that was prepared after thorough literature review and considering the local situation of the study area and purpose of the study was used to collect data. It was prepared first in English then translated to Amharic by language expert and pre-tested before final data collection.

Data were collected on socio-demographic characteristics, maternal health service uptake and infant feeding practice using recall since pregnant. In order to simplify data collection and data handling, data were collected via face to face interview technique.

#### Data quality control

Pre-test was conducted on 21 (5%) of participants at Felege Hiwot Hospital before the scheduled data collection day. Data quality was checked at the time of interview during pretest by examining consistency of data, two questions were asked at the beginning and the same questions were asked at the end. Data collectors were diploma nurse, and they had previous exposure in data collection and two days training had been given for the data collectors. The principal investigator was closely supervising the performance of the data collectors on a daily basis. Data information was checked again after data collection for completeness and its consistency.

#### Data processing and analysis

Data were first checked manually for completeness and consistency by supervisors and principal investigator during data collection and were rechecked before data entry. Data were entered and cleaned using EpiData version 3.1 then exported to SPSS version 20 for analysis. Descriptive analysis was conducted to summarize the data. Binary logistic regression analysis was executed to see the association between independent and outcome variables. All explanatory variables associated with outcome variable with p < 0.2 were entered into multivariable logistic regression analysis and significant association was identified based on p < 0.05 and AOR with 95% CI.

#### Operational definitions

*Minimum meal frequency* Feeding of infant and young children (IYC) that fulfills at least 2-3 times complementary feeding within 24 h for IYC age of 6-8 month and 3-4 times complementary feeding within 24 h for IYC age of 8 months and above [16, 17].

*Minimum dietary diversity* Feeding of IYC at least 4 food groups out of 7 food groups within 24 h (grain, legumes, dairy products, egg, meat, fruits, and vegetables) recommended by world health organization (WHO) [16, 17].

*Appropriate complementary feeding practice* Infant and young children feeding practice that fulfills the minimum dietary diversity, the minimum meal frequency, continuing breast feeding with complementary feeding for 2 years, and timely initiation of complementary feeding at the recommended time of world health organization [16, 17]..

*Inappropriate complementary feeding practice* Infant and young children feeding practice that does not fulfill even one of the components of appropriate complementary feeding practice [17].

### Results

#### Socio-demographic results

In this study, 409 mothers/care givers participated in the individual questionnaire which gives 100% response rate. From these respondents, 239 (58.4%) and 170 (41.6%) were urban and rural dwellers respectively. Two hundred twenty (53.8%) and 189 (46.2%) infants and young children were males and females respectively. Among respondents, 183 (44.7%) were in the age category of 25–30 years. Most husbands’ occupation was farmer which is 134 (32.8%) (Table 1).

**Table 1:** Socio-demographic characteristics of mothers, and infant/young children (IYC) among Debre Tabor Hospital, South Gondar Zone 2016 (n = 409)

Variables	Frequency (n = 409)	Percent (%)
Sex of child		
Male	220	53.8
Female	189	46.2
Mother’s place of residence		
Urban	239	58.4
Rural	170	41.6
Marital status		
Single	31	7.6
Married	333	81.4
Others	45	11
Religion		
Orthodox	390	95.4
Others	19	4.6
Ethnicity		
Amhara	407	99.5
Oromo	2	0.5
Educational status		
Unable to read and Write	142	34.7
Able to read, and grade 1–4	73	17.9
Grade 5–8	58	14.2
Grade 9–12	52	12.7
12+ and above	84	20.5
Number of mother’s IYC		
One	137	35.5
Two	117	28.6
Three	74	18.1
Four and above	81	19.8
Husband’s occupation		
Government employee	126	30.8
Trader	64	15.6
Non-government employee	40	9.8
Others	45	11
Maternal age		
20–30	87	21.3
31–40	183	44.7
41–50	76	18.6
Mothers’ occupation		
Farmer	136	33.3
House wife	111	27.1
Government employee	77	18.8
Others	85	20.8
Income		
< 600 Ethiopian birr	105	25.7
601–1650	118	28.9
1651–5250	165	40.3
> 5250	21	5.1
Children’s age (months)		
6–12	271	66.3
13–18	85	20.8
19–24	53	13

Others * (Marital status) = Divorced, widow, separated, cohabited

Others (occupation) = student, no job, private work

Others (Religion) = Muslims, protestant, catholic, Adventist

Others = before 6 months and after 12 months

#### Health service related results

From 409 mothers, 365 (89.2%) had ANC follow up, 326 (79.7%) delivered in health institution, 124 (30.3%) had post natal care follow up, 382 (93.4) had EPI service and 330 (80.7%) had got under five unit services. Regarding to ANC follow up, 130 (31.8%) of mothers have got only advice of exclusive breast feeding (EBF), 227 (55.5%) of respondents have got advice of optimal complementary feeding (CF) of infants/young children and 52 (12.7%) of mothers haven’t got any advice during ANC follow up.

#### Appropriate complementary feeding practice

Out of the total participants, 152 (37.2%) had practices of appropriate complementary feeding that fulfilled at least the minimum dietary diversity, the minimum meal frequency, continue breast feeding and start complementary feeding at the recommended time by world health organization(WHO). Their babies feeding frequency is proportioned as: 51 (12.5%): 8–12 times per day, 238 (58.2%): 5–7 times per day and 40 (9.8%): 3–4 times per day. Out of 409 participants, 276 (67.5%) had timely initiation of complementary feeding (Table 2).

**Table 2:** Complementary feeding (CF) practice and breast feeding related Characteristics of mothers having children age 6–24 months in Debre Tabor General Hospital, South Gondar Zone, 2016

Complementary feeding practice	Frequency (n = 409)	Percent (%)
Appropriate complementary feeding practice		
Yes	152	37.2
No	257	62.8
Food groups used for feeding per 24 h		
Grains and legumes only	231	56.5
Grains, legumes, dairy products and vegetables	64	15.6
Grains legumes, fruit, and milk	48	11.7
Grains, legumes dairy products, egg, and vegetables	40	9.8
Mothers did not remember	26	6.4
Breast feeding		
Feed IYC breast milk with CF	376	91.9
Mothers not breast feeding	33	8.1
Mothers intend to BF for 3 years and above	199	48.66
Mothers intend to BF for 2 years	177	43.3
Mother feed BM for 1 year and below	33	8.1
Frequency of feeding per 24 h		
8–12 times	51	12.5
5–7 times	238	58.2
3–4 times	40	9.8
< 2 times	80	19.6
Month of initiation of complementary feeding		
Others	30	7.3
At 6 months	276	67.5
7–8 months	70	17.1
9–12 months	33	8.1

Others = before 6 months and after 12 months

#### Factors associated with appropriate complementary feeding practice

*Bivariate analysis showed that* Maternal educational status, residence, husbands’ occupation, marital status, exclusive breast feeding practice, advice on appropriate complementary feeding practice and exclusive breast feeding during antenatal care follow up, having under 5 visits due to IYC illness and postnatal care follow up were crudely associated.

*Independently and positively associated variables in adjusted analysis were* mothers’ level of education, husbands’ occupation, exclusive breast feeding practice, advice of appropriate complementary feeding practice and exclusive breast feeding during antenatal care follow up and having under 5 visits due to IYC illness (Table 3).

**Table 3:** Bivariate and multivariate analysis of associated factors of appropriate complementary feeding practice (CFP) of mothers in Debre Tabor Hospital, South Gondar Zone 2016

Variables	Appropriate CFP		Crude OR (95% CI)	Adjusted OR (95% CI)
	Yes	No		
Educational status				
Unable to read and write	17	125	1	
Grade 1–4	17	56	2.23 (1.1, 4.7)	1.044 (0.411, 2.65)
Grade 5–8	26	32	5.97 (2.9, 12.33)	2.472 (0.99, 6.20)
Grade 9–12	31	21	10.85 (5.12, 23.0)	1.74 (0.66, 4.59)
Above grade 12	61	42	19.5 (9.71, 39.18)	2.96 (1.2, 7.62)*
Occupation of husband				
Farmer	7	127	1	
Government employee	90	36	45.36 (19.32, 106.5)	17.13 (6.04, 48.6)***
Trader	33	31	19.3 (7.8, 47.75)	8.05 (2.88, 22.51)***
Daily laborer	11	29	6.88 (2.46, 19.28)	4.01 (1.3, 12.44) *
Others	11	34	5.87 (2.12, 16.29)	4.03 (1.25, 13.01)*
EBF practice				
Yes	138	125	10.41 (5.7, 19.0)	6.12 (3.04, 12.3)***
No	14	132	1	
Antenatal care follow up				
Advice of EBF only	39	91	5.143 (1.74, 15.25)***	5.59 (1.24, 25.17)*
HE of appropriate CFP	109	118	11.1 (3.87, 31.76)***	6.34 (1.5, 26.91)*
No advice and HE	4	48	1	
Under 5 unit service				
Yes	110	220	0.44 (0.27, 0.73)***	0.44 (0.22, 0.89)*
No	42	37	1	

*P-Value ≤ 0.05, **P-Value ≤ 0.01 and ***P-Value ≤  0.001

Others = has no job, private work, students

### Discussion

In this study finding, the magnitude of appropriate complementary feeding practice was 37.2% with 95% CI of (32.8–41.5).This finding was consistent with studies’ findings of 37% in Zambia [12], 35% in Kenya [18] and 32% in South India [10]. But its finding was higher than 24.1% shown in the study done in Southern Benin [11]. The reason for this might be due to health facility service difference like health education and advice of complementary feeding practice, the study time difference and culture of the society.

Maternal educational status was one of positive indicators for appropriate complementary feeding practice (AOR = 3, 95% CI 1.2–7.62). Mothers whose educational status was above grade 12 were 3 times more likely to practice appropriate complementary feeding than those mothers who were unable to read and write. This result is similar with that of the findings in the studies done in Lahore (Pakistan), Nigeria, Nepal, Nigeria DHS, and Ethiopia [19–26]. The reason might be educated mothers have knowledge about the importance of appropriate complementary feeding practice and they could be able to assimilate advice and health education content provided during health facility contact. Additionally, educated mothers have more access of health service through having repeated contact with health facilities, communication media, and education can change the status of mothers so that they might have confidence and capacity to make decisions about their child feeding practice.

The other positively associated variable on appropriate complementary feeding practice was husbands’ occupation. Government employee (AOR = 17, CI 6.04–48.6), being trader (AOR = 8.05, CI 2.88–22.51), being daily laborer (AOR = 4.01, CI 1.3–12.44) and being others (private work, student, no job) (AOR = 4.03, CI 1.3–13.01). Being a farmer was the least to practice appropriate complementary feeding compared to above occupation types. This was similar with the findings in Nigeria and Ethiopia [23, 27]. The possible reason may be: information access difference on appropriate complementary feeding (farmers are usually with no access of information), cultural influence of male empowerment on females to make decision for appropriate complementary feeding practice (which is common among farmers) and shortage of different food staples.

Additionally, advice on exclusive breast feeding and health education/advice on appropriate complementary feeding were positive indicators with their odds of (AOR = 5.59, CI 1.24–25.17) and (AOR = 6.34, CI 1.5–26.91) respectively. This finding matches with studies in Ghana, Nepal, Nigeria, and Ethiopia [13, 21, 23, 27, 28]. The possible explanation could be: those mothers who did not get advice and health education may not have knowledge to practice appropriate complementary feeding.

There was also statistically significant (AOR = 0.44, CI 0.22–0.9) association between appropriate complementary feeding practice and getting under 5 unit service due to infant and young children illness. Mothers who visited under 5 unit due to infant and young children illness had 56% times more likely to practice appropriate complementary feeding than who did not visit under 5 unit due to child illness. This study was supported by the study done in Malaysia [4], Denmark [3], Zambia and Ethiopia [2]. This could be because of IYC illness was associated with malnutrition and malnutrition was also associated with inappropriate complementary feeding practices, so mothers going there could have got enough knowledge on how to practice the complementary feeding.

And also exclusive breast feeding practice was a positive indicator variable for appropriate complementary feeding (AOR = 6.12, CI 3.1–12.3). This finding is supported by the study done in Ethiopia [29]. It could be due to mothers who had practice of EBF are better to get health information on both EBF and complementary feeding at the same time.

## Limitation

There could be possibility of recall bias because of mothers were asked to report events of associated factors that occurred within months sometimes ago.

## Abbreviations

CF: complementary feeding
BF: breast feeding
BM: breast milk
EBF: exclusive breast feeding
CF: complementary feeding
HE: health education
